# Pre-treatment 2D and 3D dosimetric verification of volumetric arc therapy. A correlation study between gamma index passing rate and clinical dose volume histogram

**DOI:** 10.1371/journal.pone.0221086

**Published:** 2019-08-13

**Authors:** Lukasz Szczurek, Robert Juszkat, Jolanta Szczurek, Ilona Turek, Piotr Sosnowski

**Affiliations:** 1 1st Department of Medicine, Poznan University of Medical Sciences, Poznan, Poland; 2 Department of Medical Physics, International Oncology Center Affidea, Poznan, Poland; 3 Department of General and Interventional Radiology, Poznan University of Medical Sciences, Poznan, Poland; 4 Department of Diagnostic Imaging, Affidea, Poznan, Poland; 5 Department of Endocrinology, Metabolism and Internal Medicine, Poznan University of Medical Sciences, Poznan, Poland; 6 Department of Pharmacy and Biomedical Sciences, La Trobe University, Bendigo, Australia; University of Nebraska Medical Center, UNITED STATES

## Abstract

**Objectives:**

To evaluate methods for the pre-treatment verification of volumetric modulated arc therapy (VMAT) based on the percentage gamma passing rate (%GP) and its correlation and sensitivity with percentage dosimetric errors (%DE).

**Methods:**

A total of 25 patients with prostate cancer and 15 with endometrial cancer were analysed. The %GP values of 2D and 3D verifications with different acceptance criteria (1%/1 mm, 2%/2 mm, and 3%/3 mm) were obtained using OmniPro and Compass. The %DE was calculated using a planned dose volume histogram (DVH) created in Monaco’s treatment planning system (TPS), which relates radiation dose to tissue and the patient’s predicted dose volume histogram in Compass. Statistical correlation between %GP and %DE was verified using Pearson’s correlation coefficient. Sensitivity was calculated based on the receiver operating characteristics (ROC) curve. Plans were calculated using Collapsed Cone Convolution and the Monte Carlo algorithm.

**Results:**

The *t*-test results of the planned and estimated DVH showed that the mean values were comparable (*P* > 0.05). For the 3%/3 mm criterion, the average %GP was acceptable for the prostate and endometrial cancer groups, with average rates of 99.68 ± 0.49% and 99.03 ± 0.59% for 2D and 99.86 ± 0.39% and 99.53 ± 0.44% for 3D, respectively. The number of correlations was poor for all analysed data. The mean Pearson’s R-values for prostate and endometrial cancer were < 0.45 and < 0.43, respectively. The area under the ROC curve for the prostate and endometrial cancer groups, was lower than 0.667.

**Conclusions:**

Analysis of the %GP versus %DE values revealed only weak correlations between 2D and 3D verifications. DVH results obtained using the Compass system will be helpful in confirming that the analysed plans respect dosimetric constraints.

## Introduction

Volumetric modulated arc therapy (VMAT) has become a standard delivery method of intensity-modulated radiotherapy (IMRT) that improves the conformance of the dose distributions in the target area while simultaneously reducing doses in the organ at risk (OAR). As a result, organs are spared to a greater extent than that with the standard 3D conformal radiotherapy technique. In VMAT, highly conformal dose distributions are obtained through concomitant continuous gantry rotation, variable dose rate and dynamic beam modulation [1]. The increasing complexity of VMAT plans with sharp gradients requires a patient-specific quality assurance (QA). Pre-treatment verification is recommended for each VMAT plan and is essential for the detection of possible mismatches between planned and delivered doses. This process is typically performed by applying the treatment plan to a dosimetric phantom and comparing the measured and calculated phantom dose distributions based on the gamma index (GI). This method of quantitatively comparing measured and calculated dose maps was first introduced by Low et al., [2]. Detector arrays consisting of ion chambers or diodes can be used for absolute dose distribution measurement in a 2D plane or 3D geometries. In the 2D method, checking delivery precision in only a single plane exported from TPS is commonly used but is insufficient, since it is difficult to interpret the results due to missing patient anatomy. The 3D method verifies the whole patient volume, and the reconstructed dose on the CT scan is compared with the planned dose to judge dosimetric errors on their clinical relevance. A variety of VMAT verification methods have been described in the literature [3–6], some of which have been proven useful for QA; however, they have weaknesses. For instance, Electronic Portal Imaging Device (EPID) dosimetry has a limited field of view; film dosimetry offers high resolution but is labour intensive; and detector array also has a limited field of view and spatial resolution. This has led to a necessity to implement an effective quality control program [7–11] since precise delivery of the treatment equipment and calculation accuracy of the treatment planning system (TPS) must be provided to ensure that all critical aspects of the VMAT method are functioning properly.

## Materials and methods

### Patients

A total of 25 patients with prostate cancer and 15 with endometrial cancer, treated with the dual arc VMAT technique, were enrolled in the present study. Plans were optimised using the Monte Carlo (MC) algorithm in Monaco’s TPS (version 5.11.02) for 6 MV of photon energy, and were realised on an Elekta Synergy accelerator equipped with an Agility 160 multileaf collimator. Calculation options based on the dose deposition-to-medium with grid settings of 3 mm were used. In addition, statistical uncertainty (SU) for the MC algorithm was defined as 0.5% [12–13], which is a standard value in our department for radical VMAT plans. The prostate group was treated with a dose of up to 50 Gy in 25 fractions, while the planned dose for endometrial cancer patients was 45 Gy in 25 fractions. Dose evaluations were performed based on Quantitative Analyses of Normal Tissue Effects in the Clinic Group [14], International Commission on Radiation Units and Measurements [15], and Radiation Therapy Oncology Group recommendations [16–18].

### Compass system with MatriXX Evolution

The Compass software (version 3.1b) uses a 2D detector array measurement device, such as the MatriXX Evolution, which is combined with a gantry angle sensor (GAS) to measure the gantry angle [19,20]. The sensors in the MatriXX are vented pixel ionisation chambers, and each chamber has its own measurement channel. When the MatriXX is irradiated, the air in the chambers is ionised. The released charge is separated in the electrical field created between two electrodes. The current, which is proportional to the dose rate, is measured and digitised by current-sensitive analogue-to-digital converters. The chambers are arranged in a 32 x 32 grid, except for the four corner positions. The distance between the chambers is 7.62 mm from centre to centre. The effective point of measurement is 3 mm below the surface, which is 3.3 mm water equivalent depth. This level is indicated by markers on the sides of the MatriXX detector. The device is mounted to the gantry of the accelerator. The gantry mount consists of two parts: an advanced holder and a gantry fixture. The gantry fixture is customised to a type of linear accelerator (LINAC), and the advanced holder consists of an adjustable XY table and a supporting frame. The adjustable XY table is mounted on the top of a supporting frame and is used to finely adjust the MatriXX position to the crosshairs in the light field or positioning lasers of the accelerator. Geometric and absolute calibration were performed prior to measurements. Geometric calibration requires measurement of a field size larger than 7 x 7 cm, and graphical evaluation detects the edge of the field. Absolute calibration determines the response of the detector to the dose factor. In addition, a characterised Hounsfield-units-to electron density **(**HU**-**to-ED) calibration curve was implemented in the Compass system to assure accurate dose calculations on the computed tomography scan (CT). The radiotherapy plan (RT plan) from the Monaco system was exported to Compass and to the accelerator for measurement with MatriXX Evolution. In Compass, this 2D detector array measurement was used to reconstruct the fluence in four steps:

1. Computation expected fluence on the detector from a fluence model and RT plan from the TPS were exported to Compass;
2. The expected detector response for this fluence was computed based on the detector model;
3. The expected fluence was corrected using a fluence correction kernel to ensure the reconstructed fluence;
4. Dose reconstruction on CT for the reconstructed fluence was computed and compared with the Monaco dose distribution.

The Compass software provides 2D and 3D VMAT dosimetric evaluation. 3D dose distributions are presented in predicted DVH_Compass_ [21]. The reconstructed dose in the Compass system is the dose deposited in the patient’s anatomy, which is calculated from the measured detector response with a grid resolution of 3 mm, which is the same as the original planned dose from TPS.

The OmniPro and Compass systems give 1-mm resolution with linear interpolation using a low pass filter. Targets pixels are calculated by the four surrounding source pixels using a bilinear algorithm. The maximum and minimum dose rates that are detectable by the detectors are 5 Gy/min and 0.1 Gy/min, respectively. Additionally, the responses of the I’mRT MatriXX and MatriXX Evolution devices are linear with respect to dose and dose rate, but the limited resolution is insufficient to detect hot and cold spots in highly modulated VMAT plans.

**Fig 1:**
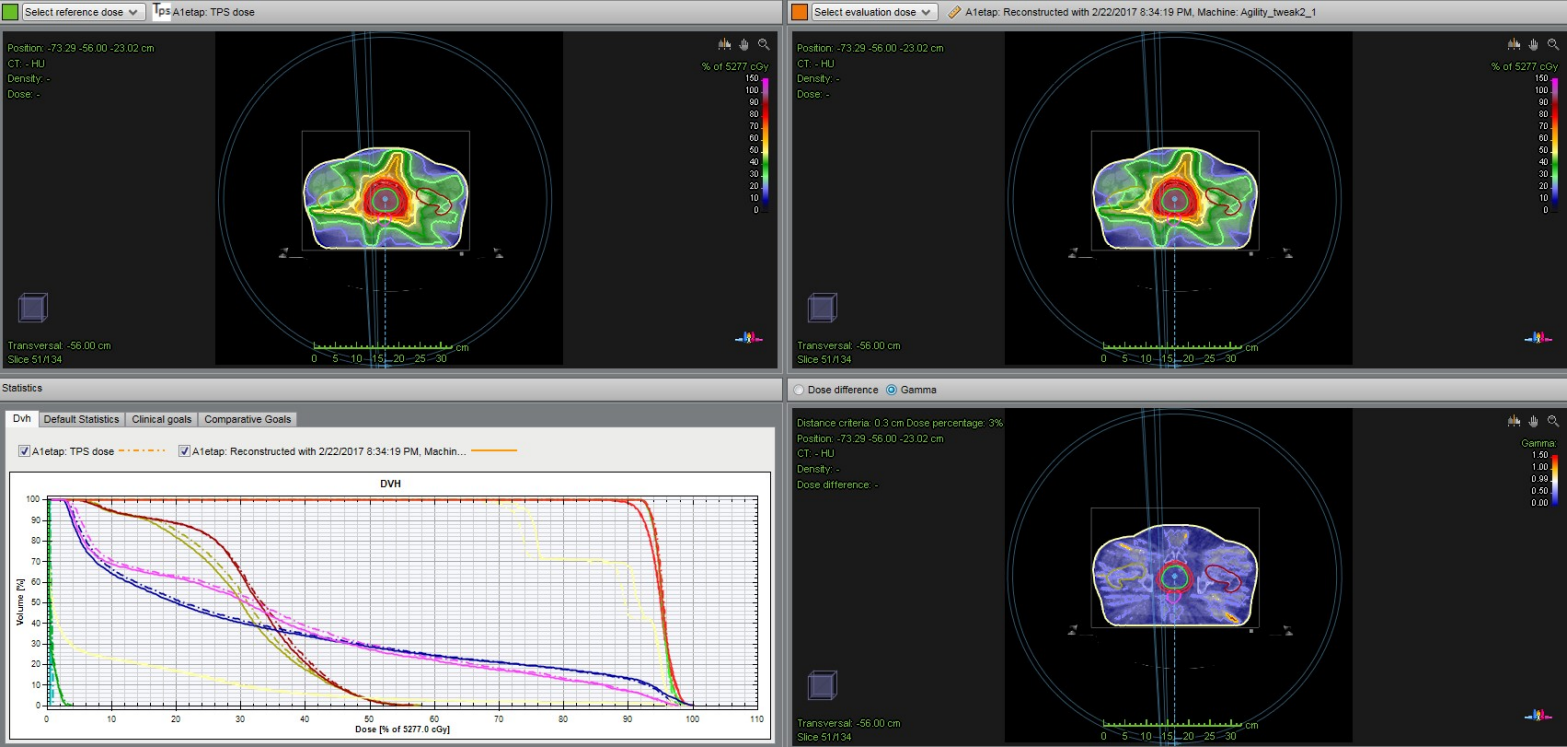
3D gamma index (GI) analysis in the Compass system on a patient’s CT scan with evaluation of DVH_Compass_ versus DVH_TPS_ in a patient with prostate cancer.

In addition, the Compass system can perform a full 3D Collapsed Cone Convolution (CCC) algorithm [22]. Fig 1 shows the 3D gamma index (GI) analysis in the Compass system.

### OmniPro system with the I’mRT MatriXX

**Fig 2:**
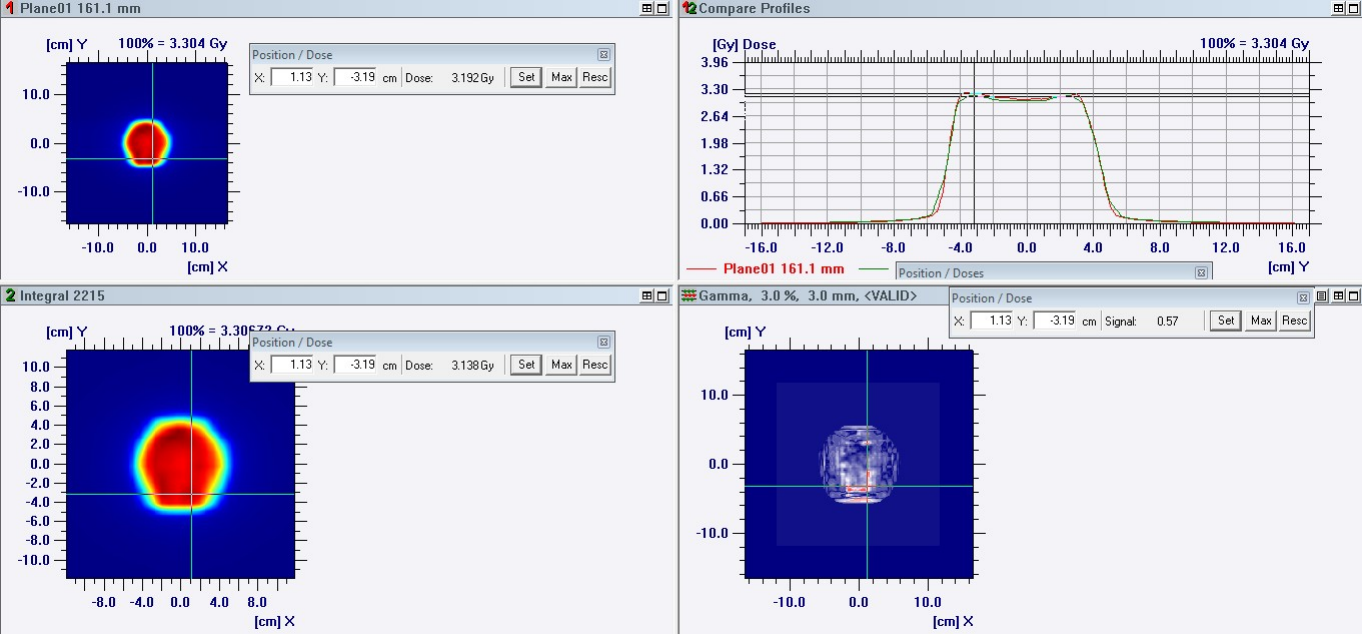
2D GI analysis in the OmniPro system. Comparison of profiles on the X- and Y-axis in patients with prostate cancer.

The OmniPro system (version 1.7b) uses the I’mRT MatriXX 2D detector array, which consists of 1020 vented ion chambers arranged in a 32 x 32 grid that resembles the Evolution array. The main difference between the I’mRT MatriXX and MatriXX Evolution is that the latter can be combined with a GAS to measure the gantry angles. The I’mRT MatriXX, with built-up and backscatter (RW3) plates, is mounted on the treatment couch under the gantry. A calibration factor was obtained prior to measurements. The system calculates k_user_ using the entered dose reference value and the average values for the four middle MatriXX chambers. A value of 100 MU, with a field of 10 x 10 cm^2^, was required during the calibration procedure. The treatment plan in the Monaco system was transferred to a measuring phantom containing the I’mRT MatriXX (QA plan). The dose plane output from the QA plan was subsequently exported to OmniPro, and measurements were then performed on a LINAC and compared with the TPS dose distribution. The OmniPro system offers 2D verification with the gamma index (GI) passing rate. Fig 2 presents the 2D GI analysis in the OmniPro system. All QA plans were delivered through MosaiQ (version 2.50, Elekta).

### Gamma analysis in 2D and 3D dosimetric verification

VMAT QA dose distributions for each treatment plan were evaluated using the GI method. The percentage gamma passing rate (%GP) was calculated for different acceptance criteria (1%/1 mm, 2%/2 mm, and 3%/3 mm) in the 2D and 3D methods of dosimetric verification in the OmniPro and Compass systems [23]. Analyses were performed with a dose threshold of 10%, and dose values below this level were not included in the comparison.

### Evaluation of the predicted dose–volume histogram

The planning target volume (PTV) parameters, D_1%_, D_98%_ (dose in 1% and 98% volume), and D_mean_, were analysed in both groups. In prostate cancer cases, D_15%_, D_25%_, D_35%_, and D_50%_ were evaluated in the bladder and rectum; D_max_, D_25%_, and D_40%_ were evaluated in the femoral head; D_mean_ was evaluated in the penile bulb and D_max_ was evaluated in the bowel. In the endometrial cancer group, D_35%_ and D_50%_ were analysed in the bladder; D_35%_, D_50%_, and D_60%_ were analysed in the rectum; D_max_ and D_15%_ were assessed in the femoral head; D_30%_ was analysed in the bowel; and D_mean_ was evaluated in the bone marrow. The percentage dosimetric errors (%DE) between the DVH values from TPS and DVH_Compass_ were calculated using:

$$\%DE=\frac{D_{DVHCompass}-D_{TPS}}{D_{TPS}}\times 100$$

where D_DVHCompass_ is the dose taken from Compass and D_TPS_ is the dose extracted from Monaco.

### Correlations and sensitivity analysis

Statistical correlation between %DE and %GP was studied using Pearson’s correlation coefficient in Statistica (version 12, StatSoft, Poland). The %GP in 2D and 3D verifications was compared with the %DE parameters from DVH for PTV and OAR. The strength of correlations, in terms of R-values, was compared. A total of 57 R-values were analysed in the prostate cancer group; 19 for each of the three acceptance criteria: 3%/3 mm, 2%/2 mm, and 1%/1 mm. The R-value was analysed for 19 dose parameters, e.g., D_1%_, D_98%_, D_mean_ in PTV; D_15%_, D_25%,_ D_35%,_ D_50%_ in bladder, etc. For the endometrial cancer group, 45 R-values were obtained (for 15 dose parameters like D_1%_, D_98%_, D_mean_ in PTV; D_35%_, D_50%_ in bladder, etc. for each of the three criteria). Numbers 19 and 15 refer to the numbers of DVH parameters evaluated for all structures from 25 patients with prostate and 15 patients with endometrial cancer, respectively, under each acceptance criterion. To quantitate the sensitivity of the GI method, the number of false negative (FN) and true positive (TP) cases were also calculated [24–26]. FN cases were included for all structures with a %DE > 3% among patients with a %GP > 95%. All cases with a %DE > 3% and a %GP < 95% were considered TP. Receiver operating characteristics (ROC) curves were generated based on the FN and TP rates, and the area under the curve (AUC) was analysed to investigate the ability of the 2D and 3D methods to accurately identify the plan with dose errors > 3%.

### CCC in the Compass system

The accuracy of the Monte Carlo algorithm in heterogeneous media was evaluated based on the secondary independent algorithm CCC. This comparison could be useful for detecting possible discrepancies in the TPS and is recommended for each treatment plan. VMAT plans from TPS were exported into the Compass system and recalculated using the CCC algorithm. Dose comparisons were performed based on the same DVH parameters used during the evaluation of the predicted DVH_Compass_ [27,28]. The percentage dosimetric errors (%DE) between the DVH values from TPS and DVH_CompassCCC_ were calculated using:

$$\%DE=\frac{D_{DVHCompass\;CCC}-D_{TPS}}{D_{TPS}}\times 100$$

where D_DVHCompassCCC_ is the dose recalculated using the CCC algorithm in Compass and D_TPS_ is the dose extracted from TPS.

## Results

### Evaluation of the %GP

The results for patients with prostate and with endometrial cancers who were treated with radiotherapy are presented in Table 1. For the 3%/3 mm criterion, the average %GP was acceptable in both the prostate and endometrial cancer groups, with an average rates of 99.68 ± 0.49% and 99.03 ± 0.59% for 2D and 99.86 ± 0.39% and 99.53 ± 0.44% for 3D, respectively. The %GP values significantly decreased with decreasing acceptance criteria. The average passing rate of the 2%/2 mm acceptance criterion was < 95% for the 2D method (OmniPro) and > 95% for the 3D method (Compass) in the endometrial cancer group. In the prostate cancer group for the same criterion, the %GP was higher than the standard action level. No patients had a %GP > 95% when using an acceptance criterion of 1%/1 mm, and the %GP was generally too low for the establishment of acceptance thresholds.

**Table 1:** The %GP between 2D and 3D VMAT QA in patients with prostate and endometrial cancers.

Acceptance criterion	Prostate cancer	Endometrial cancer
3%/3mm		
2D OmniPro	99.68 ± 0.49	99.03 ± 0.59
3D Compass	99.86 ± 0.39	99.53 ± 0.44
2D Compass	99.60 ± 0.69	97.70 ± 1.44
2%/2mm		
2D OmniPro	98.90 ± 0.74	94.89 ± 3.03
3D Compass	99.06 ± 0.82	97.42 ±1.53
2D Compass	97.75 ± 2.14	87.76 ± 5.39
1%/1mm		
2D OmniPro	94.49 ± 1.63	77.72 ± 4.46
3D Compass	93.72 ± 2.61	84.14 ± 5.40
2D Compass	75.64 ± 7.37	54.07 ± 8.84

### Dose comparison (%DE)

The %DE values obtained from TPS in Monaco and DVH_Compass_ are given in Tables 2 and 3, respectively. DVH parameters were compared using a parametric *t-*test, and *P*-values show that the doses were not significantly different (*P* > 0.05). Relatively higher %DE values were observed for parameters with a large dose gradient [29]. In the bowel structure, the %DE of D_max_ was 7.93%, which corresponds to a dose of 0.16Gy. Variable bladder filling affected the values of the standard deviations for DVH parameters in the group of patients with prostate cancer. A lower %DE for the D_1%_, D_98%_, and D_mean_ in PTV was obtained for the prostate cancer group in comparison with the gynecological group. Some differences in the bone region were as high as 3.20%.

**Table 2:** Data collection and evaluation of the %DE for prostate cancer patients treated with radiotherapy.

Structure	DVH parameter	TPS Monaco (Gy)	DVH_Compass_ (Gy)	%DE	*P*
PTV	D_1%_	51.77 ± 0.29	51.8 ± 0.46	0.08	0.75
	D_98%_	48.03 ± 0.33	48.05 ± 0.36	0.08	0.73
	D_mean_	50.09 ± 0.12	50.15 ± 0.27	0.22	0.30
Bladder	D_15%_	44.43 ± 6.60	44.40 ± 6.69	0.45	0.90
	D_25%_	36.30 ± 12.03	35.27 ±11.89	-1.49	0.89
	D_35%_	29.56 ± 14.94	28.51 ± 14.97	-2.23	0.96
	D_50%_	21.69 ± 15.29	20.32 ± 14.81	-3.62	0.95
Rectum	D_15%_	45.30 ±3.19	45.12 ± 3.44	-0.42	0.86
	D_25%_	40.50 ± 6.14	40.05 ± 6.58	-1.33	0.80
	D_35%_	35.65 ± 7.64	35.14 ± 8.04	-1.78	0.82
	D_50%_	27.81 ±9.43	27.30 ± 9.48	-2.33	0.85
Bowel	D_max_	9.84 ± 11.85	9.68 ±12.25	-7.93	0.96
Femoral head right	D_max_	22.43 ± 4.78	22.34 ± 4.90	-0.62	0.95
	D_25%_	16.14 ±3.92	15.83 ± 3.95	-2.26	0.78
	D_40%_	14.31 ± 3.60	13.97 ± 3.57	-2.65	0.75
Femoral head left	D_max_	23.76 ± 4.32	23.45 ± 4.31	-1.35	0.80
	D_25%_	16.50 ± 3.67	16.10 ±3.69	-2.52	0.71
	D_40%_	13.92 ± 3.63	13.50 ± 3.64	-3.20	0.68
Penile bulb	D_mean_	34.18 ± 13.31	34.68 ± 13.43	1.26	0.90

**Table 3:** Data collection and evaluation of the %DE for patients with endometrial cancer treated with radiotherapy.

Structure	DVH parametr	TPS Monaco (Gy)	DVH_Compass_ (Gy)	%DE	*P*
PTV	D_1%_	46.98 ± 0.17	47.08 ± 0.40	0.22	0.42
	D_98%_	42.82 ± 0.12	42.69 ± 0.24	-0.32	0.03
	D_mean_	45.05 ± 0.06	44.98 ± 0.27	-0.24	0.39
Bladder	D_35%_	43.29 ± 1.61	43.66 ± 1.32	0.88	0.51
	D_50%_	40.24 ± 2.84	40.41 ± 2.78	0.41	0.88
Rectum	D_60%_	31.02 ± 4.88	30.82 ± 4.97	-0.70	0.92
	D_50%_	34.04 ± 4.81	33.92 ± 4.93	-0.39	0.95
	D_35%_	38.92 ± 4.11	38.97 ± 4.25	0.09	0.98
Bowel	D_30%_	33.70 ± 4.59	33.76 ± 4.73	0.13	0.97
	D_10%_	43.28 ± 1.60	43.50 ± 1.74	0.69	0.64
Femoral head right	D_max_	37.44 ± 1.98	38.12 ± 2.05	1.82	0.38
	D_15%_	26.31 ± 2.42	26.33 ± 2.59	0.04	0.98
Femoral head left	D_max_	38.50 ± 1.99	39.05 ±2.22	1.42	0.49
	D_15%_	27.11 ± 2.50	27.15 ± 2.61	0.11	0.97
Bone marrow	D_mean_	25.27 ± 1.51	25.82 ± 1.51	0.96	0.86

### Correlations and sensitivity analysis

The correlations for patients with prostate and endometrial cancers are presented in Tables 4 and 5, respectively. Statistical correlations between %DE and %GP were analysed using three different acceptance criteria and three methods of dosimetric verification for DVH parameters. The number of correlations based on 3D dosimetric verifications was higher (56% of cases) than that base on the 2D dosimetric verifications in the prostate cancer group. For patients with endometrial cancer, the number of correlations based on the 3D verifications was also higher than that based on the 2D verifications (53% of cases) [24].

**Table 4:** Correlation between the 2D and 3D GI passing rate and the dose difference in patients with prostate cancer.

Acceptance criterion	Structure	DVH parameter	Correlation indices 3D	Correlation indices 2D
3%/3 mm	PTV	D_1%_	r^2^ = 0.13; r = -0.35; P = 0.28	r^2^ < 0.01; r = 0.04; P = 0.91
		D_98%_	r^2^ = 0.19; r = -0.43; P = 0.18	r^2^ = 0.03; r = -0.18; P = 0.60
		D_mean_	r^2^ = 0.59; r = -0.77; P < 0.01	r^2^ = 0.06; r = -0.24; P = 0.48
	Bladder	D_15%_	r^2^ = 0.10; r = -0.32; P = 0.34	r^2^ = 0.16; r = -0.40; P = 0.22
		D_25%_	r^2^ < 0.01; r = 0.03; P = 0.93	r^2^ = 0.04; r = -0.19; P = 0.57
		D_35%_	r^2^ = 0.09; r = -0.30; P = 0.37	r^2^ = 0.24; r = -0.49; P = 0.13
		D_50%_	r^2^ = 0.15; r = -0.39; P = 0.23	r^2^ = 0.10; r = -0.31; P = 0.35
	Rectum	D_15%_	r^2^ = 0.05; r = -0.23; P = 0.49	r^2^ = 0.07; r = -0.26; P = 0.44
		D_25%_	r^2^ < 0.01; r = -0.04; P = 0.90	r^2^ = 0.09; r = -0.30; P = 0.37
		D_35%_	r^2^ = 0.02; r = 0.13; P = 0.69	r^2^ = 0.11; r = -0.33; P = 0.32
		D_50%_	r^2^ < 0.01; r = 0.04; P = 0.90	r^2^ = 0.05; r = -0.23; P = 0.50
	Bowel	D_maks_	r^2^ = 0.19; r = -0.43; P = 0.18	r^2^ = 0.04; r = 0.20; P = 0.56
	Femoral head right	D_maks_	r^2^ = 0.04; r = 0.21; P = 0.54	r^2^ = 0.02; r = 0.15; P = 0.65
		D_25%_	r^2^ = 0.09; r = 0.30; P = 0.37	r^2^ = 0.18; r = 0.43; P = 0.19
		D_40%_	r^2^ < 0.01; r = -0.02; P = 0.96	r^2^ = 0.39; r = 0.63; P = 0.04
	Femoral head left	D_maks_	r^2^ = 0.02; r = 0.15; P = 0.65	r^2^ = 0.11; r = 0.33; P = 0.32
		D_25%_	r^2^ < 0.01; r = 0.06; P = 0.87	r^2^ < 0.01; r = 0.03; P = 0.94
		D_40%_	r^2^ = 0.22; r = 0.48; P = 0.14	r^2^ = 0.14; r = -0.38; P = 0.25
	Penile bulb	D_mean_	r^2^ < 0.01; r = -0.09; P = 0.78	r^2^ = 0.10; r = -0.31; P = 0.35
2%/2 mm	PTV	D_1%_	r^2^ = 0.20; r = -0.44; P = 0.17	r^2^ < 0.01; r = -0.07; P = 0.84
		D_98%_	r^2^ = 0.28; r = -0.53; P = 0.09	r^2^ < 0.01; r = -0.08; P = 0.82
		D_mean_	r^2^ = 0.57; r = -0.75; P < 0.01	r^2^ = 0.06; r = -0.24; P = 0.48
	Bladder	D_15%_	r^2^ = 0.56; r = -0.75; P = 0.01	r^2^ = 0.09; r = -0.30; P = 0.36
		D_25%_	r^2^ = 0.11; r = -0.33; P = 0.33	r^2^ = 0.08; r = -0.28; P = 0.41
		D_35%_	r^2^ = 0.25; r = -0.50; P = 0.12	r^2^ = 0.22; r = -0.47; P = 0.14
		D_50%_	r^2^ = 0.14; r = -0.37; P = 0.26	r^2^ = 0.03; r = -0.17; P = 0.63
	Rectum	D_15%_	r^2^ < 0.01; r = 0.05; P = 0.88	r^2^ < 0.01; r = -0.09; P = 0.79
		D_25%_	r^2^ < 0.01; r = -0.02 P = 0.95	r^2^ = 0.03; r = -0.18; P = 0.59
		D_35%_	r^2^ < 0.01; r = -0.03; P = 0.93	r^2^ = 0.04; r = -0.20; P = 0.55
		D_50%_	r^2^ < 0.01; r = 0.03; P = 0.93	r^2^ = 0.02; r = -0.14; P = 0.69
	Bowel	D_maks_	r^2^ = 0.23; r = -0.48; P = 0.14	r^2^ = 0.03; r = 0.17; P = 0.62
	Femoral head right	D_maks_	r^2^ = 0.07; r = 0.26; P = 0.44	r^2^ = 0.04; r = 0.19; P = 0.57
		D_25%_	r^2^ = 0.31; r = 0.55; P = 0.08	r^2^ = 0.12; r = 0.35; P = 0.29
		D_40%_	r^2^ = 0.15; r = 0.39; P = 0.23	r^2^ = 0.33; r = 0.58; P = 0.06
	Femoral head left	D_maks_	r^2^ = 0.09; r = 0.30; P = 0.36	r^2^ = 0.15; r = -0.39; P = 0.23
		D_25%_	r^2^ < 0.01; r = 0.05; P = 0.88	r^2^ = 0.03; r = -0.17; P = 0.61
		D_40%_	r^2^ = 0.18; r = 0.42; P = 0.19	r^2^ = 0.15; r = -0.38; P = 0.24
	Penile bulb	D_mean_	r^2^ < 0.01; r = 0.06; P = 0.86	r^2^ = 0.09; r = -0.30; P = 0.37
1%/1 mm	PTV	D_1%_	r^2^ = 0.20; r = -0.44; P = 0.17	r^2^ < 0.01; r = 0.02; P = 0.94
		D_98%_	r^2^ = 0.35; r = -0.59; P = 0.06	r^2^ = 0.02; r = 0.13; P = 0.70
		D_mean_	r^2^ = 0.65; r = -0.81; P < 0.01	r^2^ < 0.01; r = -0.05; P = 0.88
	Bladder	D_15%_	r^2^ = 0.67; r = -0.82; P < 0.01	r^2^ < 0.01; r = 0.03; P = 0.92
		D_25%_	r^2^ = 0.12; r = -0.35; P = 0.29	r^2^ = 0.02; r = -0.14; P = 0.68
		D_35%_	r^2^ = 0.14; r = -0.37; P = 0.26	r^2^ = 0.04; r = -0.21; P = 0.53
		D_50%_	r^2^ = 0.07; r = -0.26; P = 0.44	r^2^ < 0.01; r = -0.02; P = 0.96
	Rectum	D_15%_	r^2^ = 0.28; r = 0.53; P = 0.09	r^2^ = 0.02; r = -0.15; P = 0.67
		D_25%_	r^2^ = 0.26; r = 0.51; P = 0.11	r^2^ = 0.03; r = -0.16; P = -0.49
		D_35%_	r^2^ = 0.15; r = 0.39; P = 0.24	r^2^ = 0.02; r = -0.16; P = 0.65
		D_50%_	r^2^ = 0.33; r = 0.58; P = 0.06	r^2^ < 0.01; r = -0.07; P = 0.84
	Bowel	D_maks_	r^2^ = 0.10; r = -0.31; P = 0.35	r^2^ = 0.10; r = 0.32; P = 0.34
	Femoral head right	D_maks_	r^2^ = 0.25; r = 0.50; P = 0.12	r^2^ = 0.01; r = 0.12; P = 0.72
		D_25%_	r^2^ = 0.23; r = 0.48; P = 0.14	r^2^ < 0.01; r = 0.04; P = 0.90
		D_40%_	r^2^ = 0.34; r = 0.58; P = 0.06	r^2^ = 0.18; r = 0.42; P = 0.20
	Femoral head left	D_maks_	r^2^ = 0.24; r = 0.49; P = 0.12	r^2^ = 0.22; r = -0.47; P = 0.15
		D_25%_	r^2^ < 0.01; r = 0.11; P = 0.74	r^2^ = 0.07; r = -0.26; P = 0.44
		D_40%_	r^2^ = 0.03; r = 0.17; P = 0.62	r^2^ = 0.05; r = -0.22; P = 0.51
	Penile bulb	D_mean_	r^2^ = 0.03; r = 0.18; P = 0.60	r^2^ = 0.08; r = -0.29; P = 0.39

**Table 5:** Correlations between the 2D and 3D GI passing rate and dose difference in patients with endometrial cancer.

Acceptance criterion	Structure	DVH parameter	Correlation indices 3D	Correlation indices 2D
3%/3 mm	PTV	D_1%_	r^2^ = 0.67; r = −0.82; P < 0.01	r^2^ = 0.17; r = −0.41; P = 0.14
		D_98%_	r^2^ = 0.48; r = −0.69; P < 0.01	r^2^ = 0.37; r = −0.61; P = 0.02
		D_mean_	r^2^ = 0.64; r = −0.80; P < 0.01	r^2^ = 0.26; r = −0.51; P = 0.06
	Bladder	D_35%_	r^2^ < 0.01; r = −0.06; P = 0.83	r^2^ = 0.03; r = −0.18; P = 0.54
		D_50%_	r^2^ = 0.04; r = −0.19; P = 0.51	r^2^ = 0.21; r = −0.46; P = 0.10
	Rectum	D_60%_	r^2^ = 0.26; r = −0.51; P = 0.06	r^2^ = 0.13; r = −0.36; P = 0.20
		D_50%_	r^2^ = 0.20; r = −0.44; P = 0.11	r^2^ = 0.32; r = −0.56; P = 0.04
		D_30%_	r^2^ = 0.04; r = −0.19; P = 0.51	r^2^ = 0.22; r = −0.47; P = 0.09
	Bowel	D_30%_	r^2^ < 0.01; r = 0.07; P = 0.81	r^2^ = 0.39; r = −0.62; P = 0.02
		D_10%_	r^2^ = 0.24; r = 0.49; P = 0.07	r^2^ = 0.18; r = −0.42; P = 0.13
	Femoral head right	D_max_	r^2^ = 0.09; r = 0.29; P = 0.31	r^2^ = 0.08; r = −0.28; P = 0.33
		D_15%_	r^2^ < 0.01; r = −0.05; P = 0.87	r^2^ = 0.13; r = −0.36; P = 0.21
	Femoral head left	D_max_	r^2^ < 0.01; r = 0.04; P = 0.88	r^2^ = 0.01; r = −0.12; P = 0.69
		D_15%_	r^2^ < 0.01; r = 0.03; P = 0.92	r^2^ = 0.01; r = −0.10; P = 0.73
	Bone marrow	D_mean_	r^2^ = 0.62; r = −0.79; P = 0.42	r^2^ = 0.79; r = −0.89; P = 0.30
2%/2 mm	PTV	D_1%_	r^2^ = 0.71; r = −0.84 P < 0.01	r^2^ = 0.17; r = −0.42; P = 0.14
		D_98%_	r^2^ = 0.59; r = −0.77; P < 0.01	r^2^ = 0.34; r = −0.58; P = 0.03
		D_mean_	r^2^ = 0.74; r = −0.86; P < 0.01	r^2^ = 0.31; r = −0.55; P = 0.04
	Bladder	D_35%_	r^2^ = 0.03; r = 0.17; P = 0.57	r^2^ = 0.19; r = −0.43; P = 0.13
		D_50%_	r^2^ < 0.01; r = 0.05; P = 0.88	r^2^ = 0.23; r = −0.48; P = 0.08
	Rectum	D_60%_	r^2^ = 0.28; r = −0.53; P = 0.05	r^2^ = 0.03; r = −0.17; P = 0.56
		D_50%_	r^2^ = 0.21; r = −0.45; P = 0.10	r^2^ = 0.11; r = 0.34; P = 0.24
		D_30%_	r^2^ = 0.17; r = −0.42; P = 0.14	r^2^ = 0.07; r = −0.26; P = 0.36
	Bowel	D_30%_	r^2^ < 0.01; r = 0.09; P = 0.75	r^2^ = 0.45; r = −0.67; P < 0.01
		D_10%_	r^2^ = 0.13; r = 0.36; P = 0.20	r^2^ = 0.22; r = −0.47; P = 0.09
	Femoral head right	D_max_	r^2^ < 0.01; r = 0.06; P = 0.84	r^2^ = 0.03; r = −0.17; P = 0.56
		D_15%_	r^2^ = 0.01; r = 0.11; P = 0.70	r^2^ = 0.24; r = −0.49; P = 0.08
	Femoral head left	D_max_	r^2^ < 0.01; r = −0.10; P = 0.74	r^2^ = 0.01; r = −0.10; P = 0.73
		D_15%_	r^2^ < 0.01; r = −0.07; P = 0.79	r^2^ < 0.01; r = −0.04; P = 0.88
	Bone marrow	D_mean_	r^2^ = 0.16; r = 0.40; P = 0.74	r^2^ = 0.70; r = 0.84; P = 0.36
1%/1 mm	PTV	D_1%_	r^2^ = 0.68; r = −0.82; P < 0.01	r^2^ = 0.22; r = −0.47; P = 0.09
		D_98%_	r^2^ = 0.70; r = −0.84; P < 0.01	r^2^ = 0.29; r = −0.54; P = 0.05
		D_mean_	r^2^ = 0.82; r = −0.90; P < 0.01	r^2^ = 0.32; r = −0.56; P = 0.03
	Bladder	D_35%_	r^2^ = 0.06; r = 0.24; P = 0.41	r^2^ = 0.44; r = −0.66; P < 0.01
		D_50%_	r^2^ < 0.01; r = −0.07; P = 0.81	r^2^ = 0.26; r = −0.51; P = 0.06
	Rectum	D_60%_	r^2^ = 0.09; r = −0.30; P = 0.29	r^2^ = 0.01; r = −0.11; P = 0.71
		D_50%_	r^2^ = 0.06; r = −0.24; P = 0.40	r^2^ = 0.03; r = −0.17; P = 0.56
		D_30%_	r^2^ = 0.30; r = −0.54; P = 0.04	r^2^ = 0.03; r = −0.18; P = 0.54
	Bowel	D_30%_	r^2^ = 0.02; r = 0.15; P = 0.60	r^2^ = 0.27; r = −0.52; P = 0.06
		D_10%_	r^2^ = 0.09; r = 0.30; P = 0.29	r^2^ = 0.20; r = −0.45; P = 0.10
	Femoral head right	D_max_	r^2^ = 0.20; r = 0.45; P = 0.11	r^2^ = 0.02; r = −0.14; P = 0.62
		D_15%_	r^2^ = 0.20; r = 0.45; P = 0.11	r^2^ = 0.33 r = −0.57; P = 0.03
	Femoral head left	D_max_	r^2^ = 0.06; r = 0.25; P = 0.38	r^2^ < 0.01; r = −0.05; P = 0.86
		D_15%_	r^2^ = 0.20; r = 0.45; P = 0.11	r^2^ < 0.01; r = −0.06; P = 0.84
	Bone marrow	D_mean_	r^2^ = 0.20; r = −0.44; P = 0.71	r^2^ < 0.01; r = 0.06; P = 0.96

**Fig 3:**
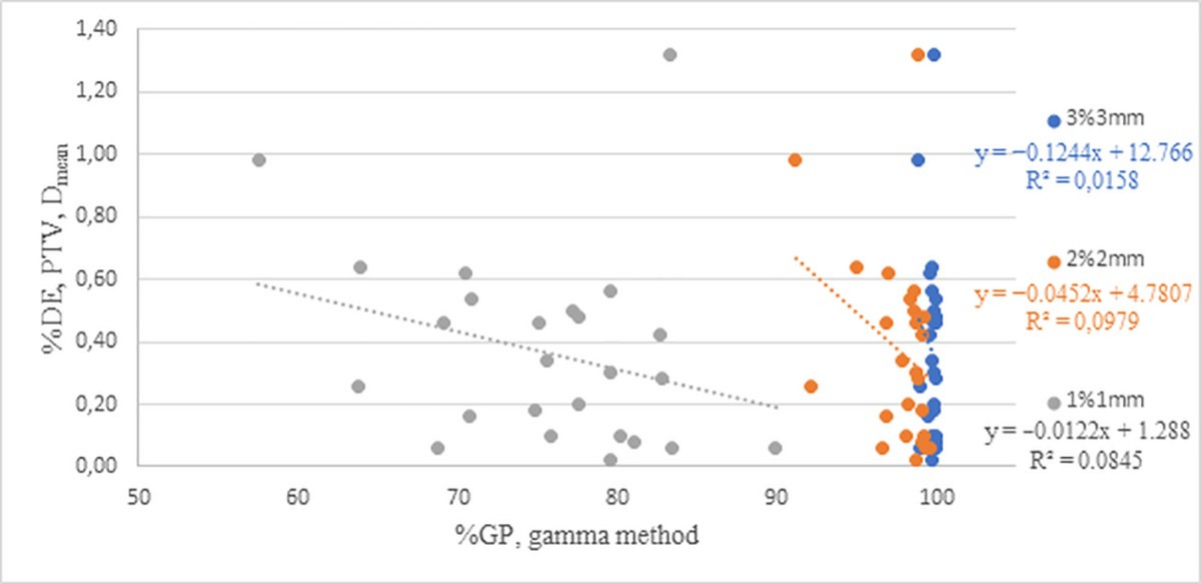
Correlation between %GP and %DE as calculated using a local method with criteria of 1%/1 mm, 2%/2 mm, and 3%/3 mm for the PTV D_mean_ in patients with prostate cancer.

The mean correlation coefficient values for patients with prostate and endometrial cancers are presented in Tables 6 and 7, respectively. The R-values were compared with the parametric *t*-test values. The *P*-values did not show any statistical difference (*P* > 0.05) in either group of patients, and the R-coefficients were mainly negative [25,30]. These values prove that there was a decrease in the clinical metrics with increasing passing rates in all treatment plans. Clinical metrics are related to DVH errors, %DE (e.g., D_1%,_ D_15%_). Negative R-values indicate a decrease in %DE parameters with increasing %GP values. The negative R-values for the 3D and 2D methods in the prostate cancer group totalled 51% and 68% of cases, respectively. Fig 3 shows the correlations between the %GP values calculated using the 3D method and the %DE for PTV in patients with prostate cancer.

**Table 6:** Mean Pearson’s correlation coefficient values between %GP and %DE for the 2D and 3D dosimetric verifications in patients with prostate cancer.

Criterion	R-value 3D	R-value 2D	*P*
3%/3 mm	0.25	0.29	0.69
2%/2 mm	0.32	0.26	0.81
1%/1 mm	0.45	0.17	0.48

**Table 7:** Mean Pearson’s correlation coefficient value between %GP and %DE for the 2D and 3D dosimetric verifications in patients with endometrial cancer.

Criterion	R-value 3D	R-value 2D	*P*
3%/3 mm	0.26	0.42	0.12
2%/2 mm	0.35	0.40	0.49
1%/1 mm	0.43	0.34	0.10

**Fig 4:**
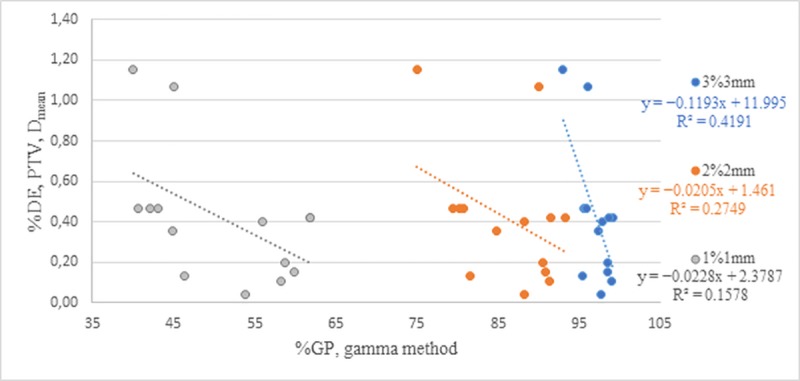
Correlation between %GP and %DE as calculated using a local method with criteria of 1%/1 mm, 2%/2 mm, and 3%/3 mm for the PTV D_mean_ in patients with endometrial cancer.

In the endometrial cancer group, the R-values were also mainly negative. The negative R-values for the 3D and 2D methods totalled 58% and 93% of cases, respectively. These results are in accordance with those previously reported [26]. In particular, for both groups and the D_mean_ parameter, the %GP calculated using the 3D method and different acceptance criteria resulted in a high correlation with %DE (r > 0.75). Fig 4 presents the correlations between %GP and %DE for PTV in the endometrial cancer group.

**Fig 5:**
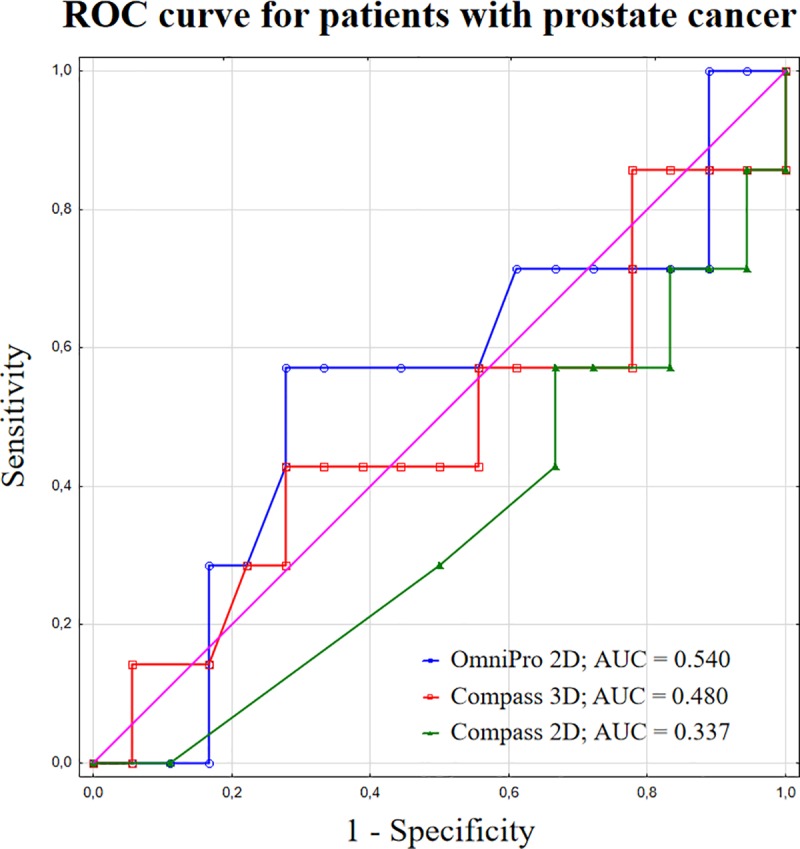
ROC curves with AUC values for patients with prostate cancer.

**Fig 6:**
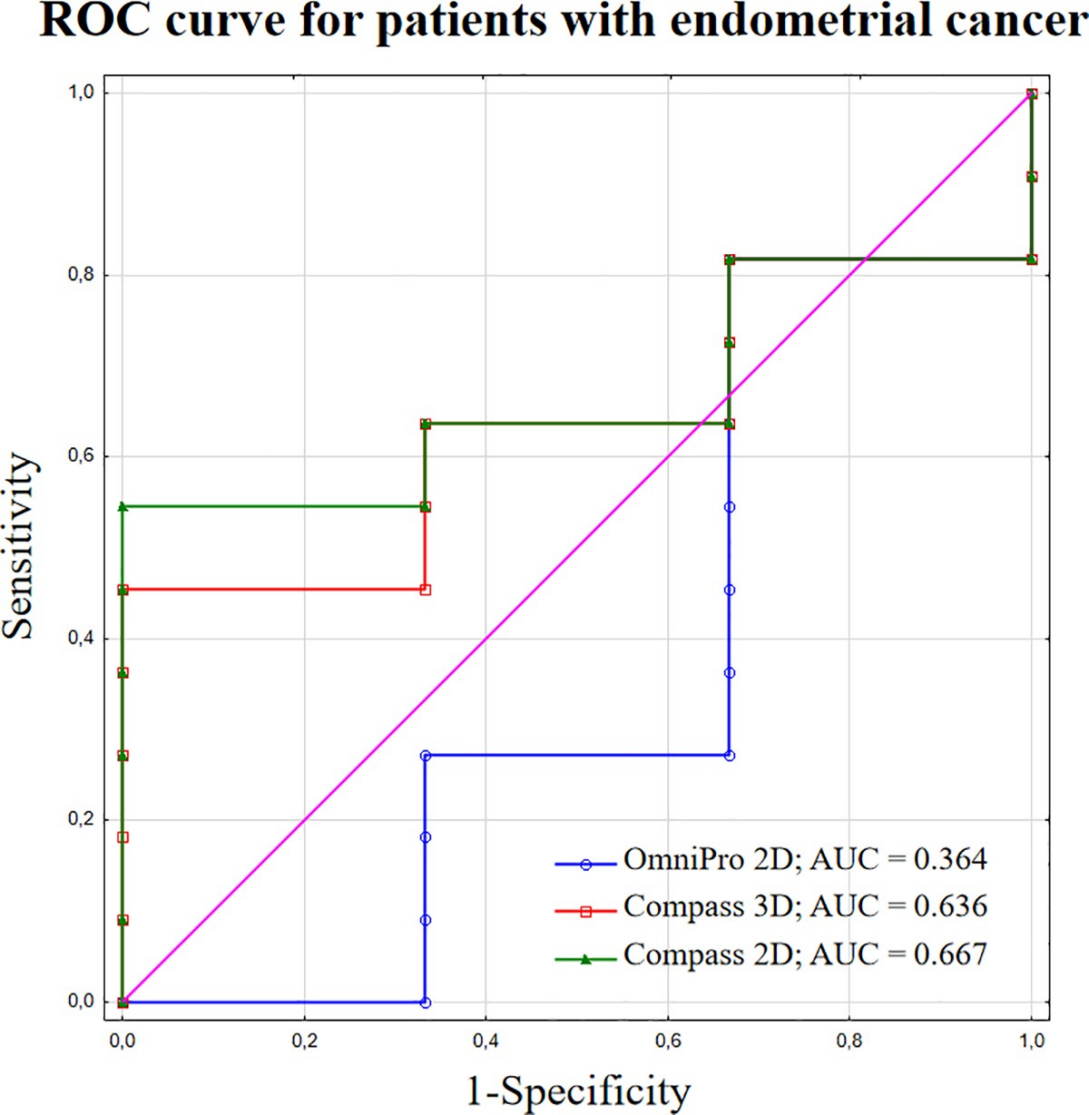
ROC curves with AUC values for patients with endometrial cancer.

Sensitivity analysis was performed for the 3%/3 mm acceptance criterion. Neither the 2%/2 mm nor 1%/1 mm level is applicable in routine clinical practice, since a %GP > 95% is difficult to achieve for all plans. The AUC values of the ROCs for DVH metrics in patients with prostate cancer were 0.540 and 0.480 for 2D and 3D, respectively. In the group of patients with endometrial cancer, the AUC values were 0.364 and 0.636 for 2D and 3D, respectively. Figs 5 and 6, presents the ROC curves for patients with prostate and endometrial cancers, respectively, with the corresponding AUC values.

### Comparison of the CCC and MC algorithms

The results of the comparison between CCC and MC in patients with prostate and endometrial cancers are given in Tables 8 and 9, respectively. The %DE in PTV was lower in the first group, but higher values were reached in the OAR with a large dose gradient.

**Table 8:** Evaluation of the %DE in patients with prostate cancer using the CCC and MC algorithms.

Structure	DVH parameter	TPS MC (Gy)	ComPass CCC (Gy)	%DE
PTV	D_1%_	51.77 ± 0.29	52.32 ± 0.38	1.07
	D_98%_	48.03 ± 0.33	48.33 ± 0.44	0.67
	D_mean_	50.09 ± 0.12	50.46 ± 0.20	0.74
Bladder	D_15%_	44.43 ± 6.60	44.27 ± 6.83	0.14
	D_25%_	36.30 ± 6.60	35.56 ± 6.83	-0.98
	D_35%_	29.56 ± 14.94	28.40 ± 14.95	-2.62
	D_50%_	21.69 ± 15.29	20.10 ± 14.69	-3.83
Rectum	D_15%_	45.30 ± 3.19	45.36 ± 3.41	0.10
	D_25%_	40.50 ± 6.14	40.24 ± 6.59	-0.85
	D_35%_	35.65 ± 7.64	35.24 ± 8.11	-1.55
	D_50%_	27.81 ± 9.94	27.27 ± 9.65	-2.83
Bowel	D_max_	9.84 ± 11.85	9.53 ± 11.98	-7.94
Femoral head right	D_max_	22.43 ± 4.78	22.46 ± 4.94	-0.10
	D_25%_	16.14 ± 3.92	16.03 ± 3.99	-0.81
	D_40%_	14.31 ± 3.60	14.15 ± 3.64	-2.65
Femoral head left	D_max_	23.76 ± 4.32	23.71 ± 4.45	-0.29
	D_25%_	16.50 ± 3.67	16.35 ± 3.75	-1.07
	D_40%_	13.92 ± 3.63	13.66 ± 3.70	-2.13
Penile Bulb	D_mean_	34.18 ± 13.31	34.19 ± 13.71	-0.75

**Table 9:** Evaluation of the %DE in patients with endometrial cancer using the CCC and MC algorithms.

Structure	DVH parameter	TPS Monte Carlo (Gy)	ComPass CCC (Gy)	%DE
PTV	D_1%_	46.98 ± 0.17	47.96 ± 0.36	2.08
	D_98%_	42.82 ± 0.12	42.91 ± 0.22	0.22
	D_mean_	45.03 ± 0.17	45.49 ± 0.36	0.97
Bladder	D_35%_	43.29 ± 1.61	43.71 ± 1.60	0.98
	D_50%_	40.24 ± 2.82	39.83 ± 4.25	0.76
Rectum	D_60%_	31.02 ± 4.88	30.82 ± 5.15	-0.76
	D_50%_	34.04 ± 4.81	33.93 ± 5.00	-0.40
	D_35%_	38.92 ± 4.11	39.06 ± 4.24	0.32
Bowel	D_30%_	33.70 ± 4.59	33.57 ± 4.62	-0.39
	D_10%_	43.28 ± 1.60	43.39 ± 1.56	0.27
Femoral head right	D_max_	37.44 ± 1.98	38.01 ± 2.22	1.50
	D_15%_	25.31 ± 2.42	26.18 ± 2.58	-0.53
Femoral head left	D_max_	38.50 ± 1.99	39.13 ± 2.09	1.63
	D_15%_	27.11 ± 2.50	27.15 ± 2.70	0.10
Bone marrow	D_mean_	25.57 ± 1.51	24.42 ± 1.59	-0.62

## Discussion

The aim of the present study was to evaluate the predictive value of GI analysis in terms of the correlation between the %GP and %DE obtained by pre-treatment QA verification. In addition, the standard action level of the 95% passing rate for 2D and 3D pre-treatment verification was analysed with the criteria of 3%/3 mm, 2%/2 mm, and 1%/1 mm. No significant differences between doses calculated using the TPS Monaco and Compass software were found for the selected DVH parameters. The pre-treatment verification was performed carefully. Analysis of the DVH results from the Compass system provided more helpful information than those from the gamma method and confirm that the analysed plans respected dose-tolerance limits. Parameters such as average dose, dose at volume, and volume at dose were more useful during the evaluation plan. Application of the gamma method for the evaluation of dose at volume may be insufficient. This can be explained by the fact that although the gamma passing rate provides the quantity of errors, it does not specify the magnitude of the error. For instance, if a 95% gamma passing rate is reported for a serial organ (e.g., the brain stem or spinal cord), what is immediately important is not whether 95% is high enough, but rather the magnitude and direction (increase or decrease) of the error for those 5% of failing voxels and their impact on the clinical relevant dose metrics (i.e., D_max_ and D_1%_) that cannot be identified from the passing rate itself. Furthermore, Nelms showed that analysis of the average GI passing rate was not acceptable on its own, since some cases with a high %GP could be clinically acceptable in one patient and unacceptable in another [31]. Therefore, evaluation based on DVH should be considered for clinical decisions.

Observed dose differences may result from incorrect implementation of irradiation or a difference between the models in the treatment planning and dosimetry systems. Possible uncertainty of treatment delivery was controlled during a nationwide audit of the IMRT technique and internal measurements. Verification of the dosimetric systems was carried out at the beginning of clinical use based on the film dosimetry. Additionally, comparison of the Compass beam modelling and OmniPro measurements with TPS using the Elekta Express QA plan was performed. The accuracy of the dose calculation model and dose delivery on the LINAC must be checked prior to clinical use.

Higher dose differences are presented for structures with a large dose gradient [29] and may result from insufficient spatial resolution of detectors used in the matrix [32]. The limited resolution of the I’mRT MatriXX and MatriXX Evolution can affect the detection of hot and cold spots in highly modulated fields. As a result, dosimetric systems may slightly underestimate or overestimate the planned dose. We observed a lower dose in the high dose region, particularly in the bowel, in the prostate group. This artifact may be caused by the interpolation of the dose measured around the ion chamber in a field with a high dose gradient. Therefore, the spatial resolution of the detector should be considered during evaluation of the measured dose. In addition, a higher %DE in bone regions was confirmed.

The %GP results fulfilled the standards recommended in the Code of Practice for QA and Control for IMRT published by the Netherlands Commission on Radiation Dosimetry [33], the European Society for Radiotherapy and Oncology [34], etc. The standards in radiotherapy recommend a pass rate > 95% using the 3%/3 mm criteria. The results obtained in the present study are in agreement with those obtained by other groups who have analysed the scores obtained from IMRT audits. The present study identified lower %GP results for 2D (OmniPro) as compared with 3D (Compass) verification. This may have been caused by the different methods of dose reconstruction in both systems, since Compass reconstructed the dose on a heterogeneous medium (CT scan of the patient) and OmniPro used a QA plan (RW3 material with a 2D detector array). For 2D analysis, the %GP pass rate decreased more rapidly than for the 3D analysis since the criteria became stricter, which is likely a result of the blurring effect, noise, or combination of both. In addition, the %GP in prostate cancer patients using the 2%/2 mm criterion was higher than the 95% action level, which should be considered in clinical practice for this group.

Relatively weak correlations between the %GP and %DE were observed for both 2D and 3D pre-treatment VMAT dosimetric evaluations. ROC curve analysis showed that the sensitivity of DVH evaluation and both GI methods was not sufficient for clinical acceptance, with AUC values < 0.667. Similar results have been previously reported in the literature [24,25,30]. Low AUC parameters confirm that the ability of 2D and 3D methods is insufficient for the accurate identification the plan with dose errors > 3%. The value of %GP shows only how many voxels fail or pass the criteria and does not provide information regarding the anatomic location of the failure or at which dose level it failed. The risk of underdosing targets and overdosing the organ at risk cannot be analysed based only on the gamma methods. Analysing the DVH results from Compass instead of the gamma passing rate gives more information about dosimetric errors and their effect on dose distribution. Therefore, the %DE obtained from pre-treatment QA verification provides a more helpful solution for VMAT QA and should be considered for clinical use.

## Conclusions

The present study identified weak correlations and sensitivity between the GI passing rate and dose errors from the dose–volume histograms for 2D and 3D pre-treatment verifications. The %GP only shows how many voxels failed to pass the criteria and is insufficient for the evaluation of dose parameters; therefore, the gamma passing rate cannot be exclusively relied upon. Evaluation of the clinical tolerance of PTV and OAR should be implemented. Comparison of the CCC and MC algorithms in the pelvic region led to similar results and may be useful for detecting possible discrepancies in the TPS [35,36]. The results indicate that the percentage dose difference between the Compass software and the TPS calculation was <2.09% for analysis using the definition of D_1%_, D_98%_, and D_mean_ in PTV for each group.

New approaches to evaluate QA plans need to be urgently implemented in clinical practice. VMAT QA analysis with a methodology that allows clinicians to predict the impact of a delivered dose on the DVH curve from 3D reconstructions of patient anatomy needs to be employed.
